# Metformin protects primary rat hepatocytes against oxidative stress-induced apoptosis

**DOI:** 10.1002/prp2.125

**Published:** 2015-03-18

**Authors:** Laura Conde de la Rosa, Titia E Vrenken, Manon Buist-Homan, Klaas Nico Faber, Han Moshage

**Affiliations:** Department of Gastroenterology and Hepatology, University Medical Center Groningen, University of GroningenGroningen, The Netherlands

**Keywords:** Apoptosis, cell death, cell signaling, hepatocytes, hepatotoxicity, insulin, JNK, metformin, oxidative stress, superoxide anions

## Abstract

The majority of chronic liver diseases are accompanied by oxidative stress, which induces apoptosis in hepatocytes and liver injury. Recent studies suggest that oxidative stress and insulin resistance are important in the pathogenesis of nonalcoholic fatty liver disease (NAFLD) and the pathophysiology of diabetes complications. Metformin has been shown to be hepatoprotective in the insulin-resistant and leptin-deficient ob/ob mouse model of NAFLD. However, the mechanism involved in the protective effects of metformin has not been elucidated yet. Therefore, we investigated the protective effect of metformin against oxidative stress-induced apoptosis. Primary rat hepatocytes were exposed to the oxidative stress-generating compound menadione in the presence and absence of metformin. Apoptosis was determined by measuring caspase activity and poly(ADP-ribose) polymerase (PARP)-cleavage, and necrosis was measured by Sytox Green nuclear staining. We demonstrate that (1) Metformin inhibits menadione-induced caspase-9,-6,-3 activation and PARP-cleavage in a concentration-dependent manner. (2) Metformin increases menadione-induced heme oxygenase-1 (HO-1) expression and inhibits c-Jun N-terminal kinase (JNK)-phosphorylation. (3) Metformin does not induce necrosis in primary hepatocytes. Metformin protects hepatocytes against oxidative stress-induced caspase activation, PARP-cleavage and apoptosis. The anti-apoptotic effect of metformin is in part dependent on HO-1 and bcl-xl induction and inhibition of JNK activation and independent of insulin signaling. Our results elucidate novel protective mechanisms of metformin and indicate that metformin could be investigated as a novel therapeutic agent for the treatment of oxidative stress-related liver diseases.

## Introduction

Most chronic liver diseases are accompanied by oxidative stress (Bomzon et al. 1997; Reid 2001; Loguercio and Federico 2003), which may induce cell death (apoptosis and/or necrosis) in hepatocytes, leading to liver injury. Apoptosis, or programmed cell death (Nieminen 2003; Nikoletopoulou et al. 2013; Stevens et .al. 2013), is an active process characterized by cell shrinkage, chromatin condensation, and activation of caspases (Riedl and Shi 2004; Shi 2002), and formation of apoptotic bodies. In contrast, necrosis is associated with ATP depletion, rupture of the plasma membrane, and spilling of the cellular content eliciting inflammation (Nieminen 2003; Nikoletopoulou et al. 2013; Stevens et al. 2013). Although necrosis has traditionally been considered passive cell death as opposed to apoptotic or programmed cell death, there is increasing evidence that necrotic cell death is also dependent on active intracellular signaling pathways and that some of these signaling pathways are shared with apoptotic cell death, for example, the mitochondrial permeability transition (Samali et al. 1999; Nieminen 2003; Yuan and Kaplowitz 2009; Jaeschke et al. 2012; Nikoletopoulou et al. 2013; Stevens et al. 2013). To underscore the fact that necrosis and apoptosis are actually two sides of the same coin, the term necroapoptosis has been adopted and it has been described that cells can switch between these modes of cell death, depending on specific conditions, for example, intracellular ATP levels and redox status (Samali et al. 1999; Nieminen 2003; Yuan and Kaplowitz 2009; Jaeschke et al. 2012; Nikoletopoulou et al. 2013; Stevens et al. 2013). Recent studies suggest that oxidative stress and insulin resistance are important in the pathogenesis of nonalcoholic fatty liver disease (NAFLD) and the pathogenesis of complications of diabetes (Maechler and Wollheim 2001; Rosen et al. 2001; Du et al. 2003; Evans et al. 2003; Wiernsperger 2003; Marchetti et al. 2004; Bugianesi et al. 2005; Machado and Cortez-Pinto 2005).

Metformin, an antihyperglycemic agent used in type 2 diabetes, has been shown to have beneficial effects in many studies (Mataforme et al. 2011; Barbero-Becerra et al. 2012; Cicero et al. 2012; Mazza et al. 2012). In addition, metformin has been shown to be hepatoprotective in the insulin-resistant and leptin-deficient ob/ob mouse model of NAFLD (Lin et al. 2000). Metformin also appears to have beneficial effects on (liver) diseases not directly related to insulin resistance, including tumorigenesis (Cicero et al. 2012; Kita et al. 2012; Quinn et al. 2013). In nondiabetic experimental animal models, metformin prevents endotoxin-induced liver injury after partial hepatectomy (Bergheim et al. 2006) and protects against carbon tetrachloride-induced hepatotoxicity in mice (Poon et al. 2003). Furthermore, metformin has been shown to protect endothelial cells and neuronal cells in several in vitro toxicity models, possibly related to protection against oxidative stress (El-Mir et al. 2008; Eriksson et al. 2012; Bhatt et al. 2013). However, the mechanism(s) involved in these protective effects of metformin has not been elucidated yet.

Since metformin may have protective effects, in particular against oxidative stress, that are not related to improving insulin-resistance, we investigated whether metformin has any protective effect against oxidative stress-induced apoptosis in primary rat hepatocytes. We demonstrate that metformin protects primary rat hepatocytes against oxidative stress-induced apoptosis. The protective effect of metformin is in part dependent on the induction of heme oxygenase-1 (HO-1) and bcl-xl and inhibition of c-Jun N-terminal kinase (JNK) activation, and is independent of insulin signaling.

Hence, metformin could represent an important therapeutic agent for liver diseases associated with excessive oxidative stress.

## Materials and Methods

### Animals

Specified pathogen-free male Wistar rats (220–250 g) were purchased from Harlan (Zeist, the Netherlands). They were housed under standard laboratory conditions with free access to standard laboratory chow and water. Experiments were performed following the guidelines of the local Committee for Care and Use of laboratory animals.

### Rat hepatocyte isolation

Rat hepatocytes were isolated as described previously (Moshage et al. 1990) and cultured in William's E medium (Invitrogen, Breda, the Netherlands) supplemented with 50 *μ*g/mL gentamicin (Invitrogen) without the addition of insulin or growth factors. During the attachment period (4 h), 50 nmol/L dexamethasone (Sigma, St. Louis, MO, USA), and 5% fetal calf serum (Invitrogen) were added to the medium. After the attachment period of 4 h, cells were washed and cultured in serum-free medium without insulin or dexamethasone. Cells were cultured in a humidified incubator at 37°C and 5% CO_2_. Hepatocyte viability was always more than 90% and purity more than 95% as determined by Trypan blue exclusion assay. All the experiments were performed in the absence of insulin.

### Experimental design

Experiments were started 24 h after isolation of hepatocytes (20 h after attachment). At this time point, it was assumed that no effects of insulin or dexamethasone are present. As a model of oxidative stress-induced apoptosis, we used menadione (2-methyl-1,4-naphthoquinone). The quinone menadione undergoes one- electron reduction catalyzed by NADPH-cytochrome P-450 reductase or NADH ubiquinone oxidoreductase, yielding a semiquinone reactive intermediate that undergoes redox cycling to generate superoxide anions (O_2_^•−^) (Thor et al. 1982). This model has been described before and induces predominantly apoptotic cell death in primary cultures of rat hepatocytes (Conde de la Rosa et al. 2006). Cells were exposed to menadione (50 *μ*mol/L; Sigma) for the indicated time and/or metformin (0.1–0.5 mmol/L), added 10 min before menadione treatment. The concentrations of metformin used were based on our previous study in which the protective effect of metformin on bile acid-induced apoptosis was described and represent the two lowest effective concentrations of metformin against menadione-induced apoptosis (Woudenberg-Vrenken et al. 2013). In some experiments, the insulin-sensitizing drug rosiglitazone (0.5–10 *μ*mol/L) and insulin (50 nmol/L) was used.

### Caspase enzyme activity assays

Caspase-3 enzyme activity was assayed as described previously (Schoemaker et al. 2004). Caspase-6 activity was assayed according to the manufacturer's instructions (BioVision, Milpitas, CA, USA).

### Sytox green nuclear staining

Rupture of the plasma membrane distinguishes necrotic from apoptotic cell death. To estimate necrotic cell death, hepatocytes were incubated 15 min with Sytox green (Molecular Probes, Eugene, OR) nucleic acid stain, which penetrates cells with compromised plasma membranes but does not cross the membranes of viable cells or apoptotic bodies. Fluorescent nuclei were visualized using an Olympus CKX41 microscope at 450–490 nm. Hepatocytes exposed to 5 mmol/L hydrogen peroxide (H_2_O_2_) were used as a positive control for necrosis (Conde de la Rosa et al. 2006).

### Western blot analysis

Western blot analysis of cell lysates was performed by SDS-PAGE (sodiumdodecyl sulphate polyacrylamide gel electrophoresis) followed by transfer to Hybond ECL nitrocellulose membrane (Amersham - GE Electric, Eindhoven, The Netherlands). An antibody against glyceraldehyde 3-phosphate dehydrogenase (GAPDH) (Calbiochem, San Diego, CA, USA) and Ponceau S staining were used to ensure equal protein loading and electrophoretic transfer.

Caspase cleavage was detected using polyclonal rabbit antibodies against cleaved caspase-9, -6, and -3. Poly(ADP-ribose) polymerase (PARP) cleavage was detected with a rabbit anti-PARP polyclonal antibody (Cell Signaling, Beverly, MA). PARP (116 kDa) is a substrate of caspase-3 yielding a product of 89 kDa and it is considered a late marker for apoptosis. Phosphorylation of IRS-1 (insulin receptor substrate-1) was determined using an antibody against Ser612-phosphorylated IRS-1. After western blot analysis for p-JNK (monoclonal; Santa Cruz Biotechnology, Dallas, TX, USA), blots were stripped using 0.1% SDS in PBS/Tween-20 at 65°C for 30 min and incubated with antibodies against total-JNK. Antibodies against p-JNK and phosphorylated IRS-1 were obtained from Cell Signaling Technology and used at 1:1000 dilution. Western blot for phosphorylated IRS-1 was quantified by densitometry using the Biorad PhotoDoc system and software (Image Lab vs. 3.0: Belgrade, MT, USA).

### RNA isolation and quantitative real-time PCR

RNA was isolated using the Tri-reagent (Sigma) according to the manufacturer's instructions Reverse transcription was performed on 2.5 *μ*g of total RNA using random primers in a final volume of 50 *μ*L (reverse transcription system; Sigma). Real-time detection was performed on the ABI PRISM 7700 (PE Applied Biosystems - Thermo Fisher Scientific, Breda, The Netherlands) initialized by 10 min at 95°C, followed by 40 cycles of 15 sec at 95°C, and 1 min at 60°C. Each sample was analyzed in duplicate. 18S mRNA levels were used as an endogenous control. Real-time PCR primers and probes for HO-1 (Conde de la Rosa et al. 2008): Forward: CAC AGG GTG ACA GAA GAG GCT AA; Reverse: CTG GTC TTT GTG TTC CTC TGT CAG; Probe: CAG CTC CTC AAA CAG CTC AAT GTT GAG C and for Bcl-xl: Forward: TTG TGG ATC TCT ACG GGA ACA AT; Reverse: GTC AGG AAC CAG CGG TTG AA; Probe: CTC CTG GCC TTT CCG GCT CTC G.

### Statistical analysis

All data are expressed as the mean of 3–5 independent experiments ± SD. Statistical significance was determined by the Mann–Whitney test; *P* < 0.05 was considered statistically significant.

## Results

### Metformin but not rosiglitazone protects against menadione-induced caspase activation, PARP cleavage, and apoptosis

**Figure 1:**
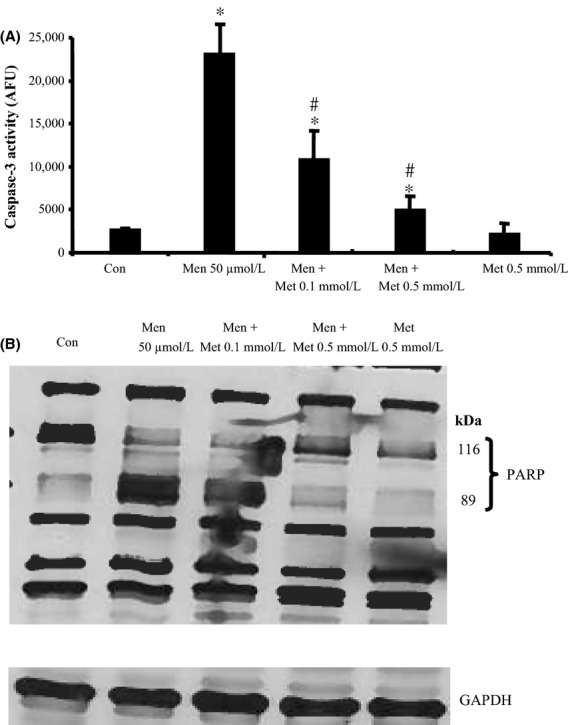
Metformin prevents oxidative stress-induced apoptosis and PARP cleavage in a concentration-dependent manner. Primary hepatocytes were exposed to metformin (Met, 0.1 mmol/L and 0.5 mmol/L) 10 min before and during menadione (Men, 50 *μ*mol/L, 9 h) treatment. Control (con, untreated cells). (A) Caspase-3 activity assay. (B) Western blot was performed against native (116 kDa) and cleaved (89 kDa) PARP. GAPDH was used as a loading control. Statistical analysis: *P < 0.05 compared to control. #*P* < 0.05 compared to menadione-treated cells; *n* = 5. PARP, poly(ADP-ribose) polymerase.

We investigated whether metformin exerts a protective effect against oxidative stress-induced apoptosis. As shown in Figure1A, metformin inhibits oxidative stress-induced apoptosis (expressed as caspase-3 activity) in primary hepatocytes. The protective effect of metformin reached its maximum at 0.5 mmol/L. In addition, metformin inhibits menadione-induced PARP-cleavage, which is considered a late marker for apoptosis (Fig.1B), suggesting a protective role of metformin against oxidative stress-induced apoptosis.

**Figure 2:**
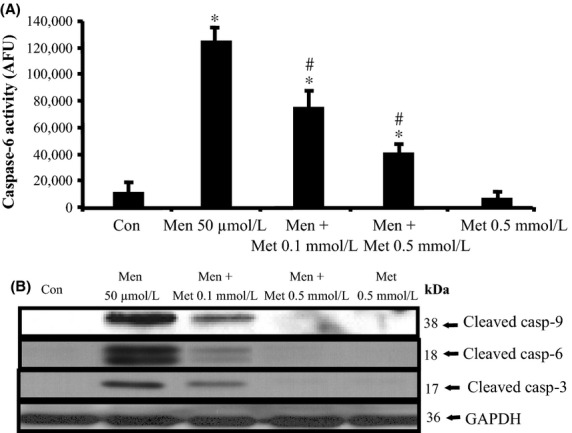
Metformin blocks menadione-induced caspase-9, -6 and -3 activation in a dose-dependent manner in primary rat hepatocytes. Cells were incubated with the superoxide anion–donor menadione (Men, 50 *μ*mol/L, 9 h) or not (control, untreated cells) in the presence and absence of metformin (Met, 0.1 mmol/L and 0.5 mmol/L, added 10 min before and during menadione treatment). (A) Caspase-6 activity assay: Metformin dose-dependently prevents caspase-6 activation. (B) Western Blot: Metformin inhibits caspase-9, -6, and -3 processing and cleavage induced by the superoxide anion-donor menadione in a concentration-dependent manner. GAPDH was using as a loading control. Statistical analysis: *P < 0.05 compared to control. #*P* < 0.05 compared to menadione-treated cells; *n* = 5. GAPDH, glyceraldehyde 3-phosphate dehydrogenase.

In previous studies, we showed that menadione at the concentration used, induces predominantly caspase-dependent apoptotic cell death but hardly any necrotic cell death in primary rat hepatocytes (Conde de la Rosa et al. 2006, 2008). In these studies, we also demonstrated activation of caspases-3, 6, and 9 in menadione-induced apoptosis in rat hepatocytes. Therefore, we analyzed whether metformin has an effect on the activation of these caspases. Metformin prevents menadione-induced capase-6 activity (Fig.2A) and caspase-9, -6, and -3 cleavage in primary rat hepatocytes (Fig.2B).

**Figure 3:**
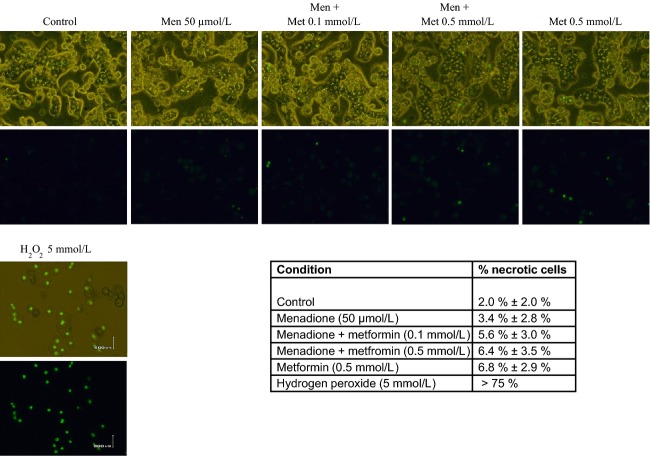
Metformin does not induce necrotic cell death in primary hepatocytes. Cells were incubated with menadione (Men, 50 *μ*mol/L, 9 h) and without (Con, untreated cells) in the presence and absence of metformin (Met, 0.1 mmol/L and 0.5 mmol/L, added 10 min before menadione exposure). Sytox Green nucleic staining: necrotic cells represent less than 2% from total cells in all conditions tested. Hepatocytes exposed to 5 mmol/L hydrogen peroxide was used as positive control for necrosis. Upper panel phase contrast and fluorescence micrographs, lower panel fluorescence micrographs only. Magnification 40x. Percentages of necrotic cells represent means ± SEM. Percentages were calculated by counting total number of cells and necrotic cells (fluorescent nuclei) in 3 randomly chosen fields in 3 independent experiments.

**Figure 4:**
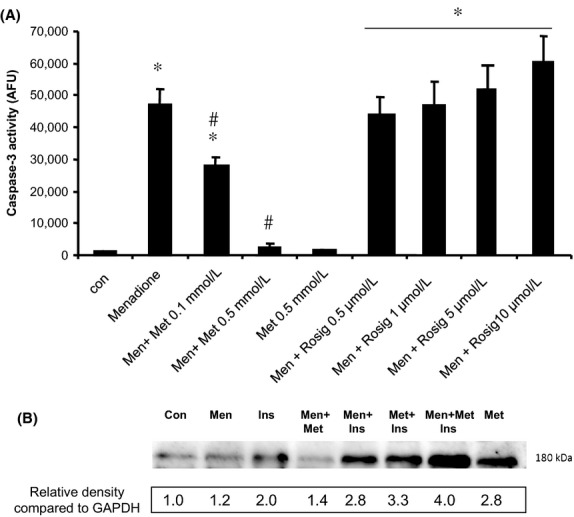
(A) Rosglitazone is not protective against menadione-induced apoptosis in hepatocytes. Cells were incubated with menadione (Men, 50 *μ*mol/L, 9 h) and without (Con, untreated cells) in the presence and absence of the insulin sensitizing compound rosiglitazone (Rosig, 0.5–10 mmol/L). *P < 0.05 compared to control. #*P* < 0.05 compared to menadione-treated cells. (B) Insulin-induced phosphorylation of IRS-1 is slightly but significantly increased by metformin but not modulated by menadione. Densitometric values of bands are expressed relative to GAPDH with the value of GAPDH in the control condition set as 1. Hepatocytes were exposed to 50 nmol/L insulin in the presence and absence of 0.5 mmol/L metformin or 50 *μ*mol/L menadione or their combination. Menadione and metformin were added 3 h prior to addition of insulin and cells were harvested for Western blot analysis 5 min after insulin exposure. IRS-1, insulin receptor substrate-1. GAPDH, glyceraldehyde 3-phosphate dehydrogenase.

In addition, metformin did not induce significant necrosis in primary hepatocytes (Fig.3), indicating that metformin is protective against oxidative stress-induced apoptosis and does not switch the balance between apoptosis and necrosis. Since metformin has been reported to partially reverse the insulin-resistance in diabetes mellitus type 2, we also investigated the effect of the insulin-sensitizing drug rosiglitazone on oxidative stress-induced apoptosis. Rosiglitazone up to 10 *μ*mol/L did not protect against oxidative stress-induced apoptosis, but rather increased oxidative stress-induced apoptotic hepatocyte death (Fig.4A). Moreover, insulin at 50 nmol/L did not inhibit menadione-induced hepatocyte apoptosis (data not shown). Finally, we investigated whether metformin and/or menadione have any effect on insulin-signaling, using IRS-1 as a downstream target of insulin. As shown in Figure4B, insulin-induced phosphorylation of IRS-1. Insulin-induced phosphorylation of IRS-1 was not modulated by menadione. Metformin alone or in the presence of insulin slightly but significantly increased phosphorylation of IRS-1.

### Metformin reduces oxidative stress-induced JNK phosphorylation

We have previously demonstrated that oxidative stress-induced hepatocyte death is dependent on JNK activation, since menadione activates JNK and inhibition of JNK activity abolished oxidative stress-induced apoptosis (Conde de la Rosa et al. 2004; 2008) Therefore, we examined the effect of metformin on menadione-induced JNK activation.

**Figure 5:**
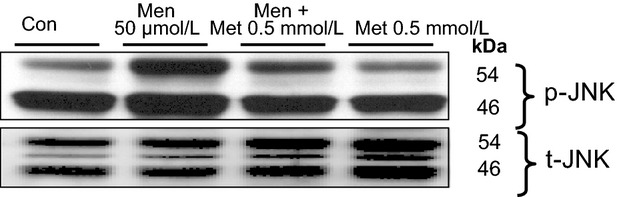
Metformin prevents oxidative stress-induced JNK phosphorylation in primary hepatocytes. Cells were exposed to menadione (Men, 50 *μ*mol/L, 1 h) or not (control, untreated cells) in the presence and absence of metformin (Met, 0.5 mmol/L, added 10 min before menadione treatment). Western blots were performed against phosphorylated-JNK (p-JNK) and total-JNK (t-JNK, used as loading control).

In the current study, metformin does not induce JNK-phosphorylation, but reduces menadione-induced JNK-phosphorylation (Fig.5). The residual JNK-phosphorylation observed in control hepatocytes is most likely the result of a basal phosphorylation due to isolation and culture manipulations. Interestingly, the inhibitory effect of metformin appeared to be stronger on the JNK p54 isoforms than the p46 isoforms. This is similar to the effect of the HO-1 product carbon monoxide on JNK-phosphorylation, as reported previously (Conde de la Rosa et al. 2008). These results suggest that the protective effect of metformin against oxidative stress-induced apoptosis is in part via inhibition of JNK (p54) activation.

### Metformin induces menadione-induced HO-1 expression

**Figure 6:**
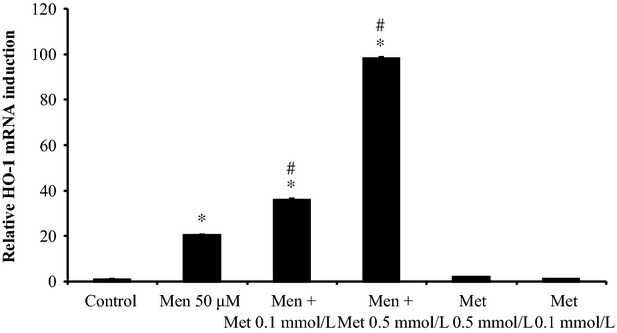
Metformin increases menadione-induced HO-1 upregulation in a concentration-dependent manner. Quantitative real-time PCR: cells were incubated with different concentrations of metformin (Met, 0.1 mmol/L and 0.5 mmol/L, 9 h), added 10 min before the addition of menadione (Men, 50 *μ*mol/L, 9 h). Statistical analysis: *P < 0.05 compared to control. #*P* < 0.05 compared to menadione-treated cells; *n* = 3. HO-1, heme oxygenase-1.

HO-1 has been described to be cytoprotective against oxidative stress. Previously, we showed that the superoxide anion donor menadione induces HO-1 expression in a time and concentration-dependent manner, although not sufficiently to prevent apoptosis. However, we demonstrated that overexpression of HO-1, using the adenovirus Ad5HO-1, leads to protection against oxidative stress-induced apoptosis in primary hepatocytes (Conde de la Rosa et al. 2008). This prompted us to investigate whether metformin has any effect on HO-1 expression. As shown in Figure6, metformin further increases menadione-induced HO-1 expression, although metformin alone does not induce HO-1 expression in primary hepatocytes.

### Metformin increases menadione-induced bcl-xl expression

**Figure 7:**
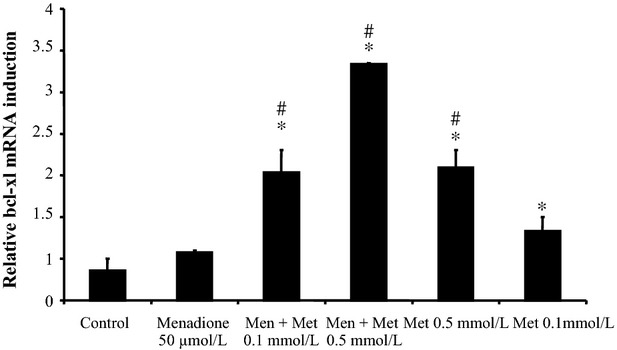
Metformin induces bcl-xl expression in a concentration-dependent manner. Quantitative real-time PCR: cells were incubated with metformin (Met, 0.1 mmol/L and 0.5 mmol/L, 9 h), added 10 min before menadione treatment (Men, 50 *μ*mol/L, 9 h). *P < 0.05 compared to control. #*P* < 0.05 compared to menadione-treated cells; *n* = 3.

HO-1 induction has been demonstrated to correlate with increased expression of the anti-apoptotic bcl-2-family member bcl-xl (Goodman et al. 2007; Banerjee et al. 2012). Since we have previously shown that bcl-xl is expressed in hepatocytes (Schoemaker et al. 2002), we investigated the effect of oxidative stress and metformin on bcl-xl expression. As shown in Figure7, metformin further promoted menadione-induced bcl-xl expression. Notably, metformin alone dose dependently increases bcl-xl expression in primary rat hepatocytes.

## Discussion

Many studies have shown that metformin has beneficial effects in the treatment of type 2 diabetes (Inzucchi et al. 1998; Knowler et al. 2002; Mataforme et al. 2011; Cicero et al. 2012), a disorder that has been associated with oxidative stress (Maechler and Wollheim 2001; Rosen et al. 2001; Du et al. 2003; Evans et al. 2003; Wiernsperger 2003; Marchetti et al. 2004; Machado and Cortez-Pinto 2005). In addition, metformin has been demonstrated to be hepatoprotective in models of NAFLD, a condition also known to be associated with oxidative stress (Lin et al. 2000; Poon et al. 2003; Bergheim et al. 2006; Mataforme et al. 2011; Barbero-Becerra et al. 2012; Cicero et al. 2012). Although an important part of the beneficial effects of metformin has been attributed to its (partial) reversal of insulin-resistance, there are also reports describing beneficial effects of metformin in nondiabetic disorders and experimental models, in particular suggesting a protective effect of metformin against oxidative stress (Poon et al. 2003; Bergheim et al. 2006; El-Mir et al. 2008; Eriksson et al. 2012; Kita et al. 2012; Bhatt et al. 2013; Quinn et al. 2013).

In this study, we document that metformin protects primary rat hepatocytes against oxidative stress-induced apoptosis, determined by caspase-9, -6 and -3 activation, the caspases most intimately involved in menadione-induced apoptosis of rat hepatocytes, and PARP cleavage in a concentration-dependent manner. Our results are in accordance with recent studies, in which metformin prevents high glucose-induced endothelial and neuronal cell death, at least in part by inhibiting the mitochondrial permeability transition and/or inhibition of ER stress, resulting in inhibition of pro-apoptotic JNK activation (Detaille et al. 2005; El-Mir et al. 2008; Eriksson et al. 2012; Jung et al. 2012; Bhatt et al. 2013) and ameliorates functional defects, activation of caspases and apoptosis in pancreatic islets from type 2 diabetic patients (Marchetti et al. 2004). Furthermore, in a recent study, we demonstrated that metformin protects hepatocytes against bile acid-induced apoptosis, but not against TNF*α*-induced hepatocyte apoptosis (Woudenberg-Vrenken et al. 2013).

Moreover, metformin inhibits caspase-9 activation, suggesting that metformin prevents disruption of mitochondria and subsequent apoptosis (Detaille et al. 2005; El-Mir et al. 2008; Bhatt et al. 2013). In endothelial cells, it has been shown that metformin blocks the mitochondrial permeability transition and cytochrome c release induced by high glucose-derived oxidative stress in endothelial cells (Detaille et al. 2005).

In a previous study, we demonstrated that apoptotic cell death can be switched to necrotic cell death under certain conditions (Conde de la Rosa et al. 2006). In the present report, we provide evidence that metformin indeed protects hepatocytes exposed to oxidative stress and does not switch apoptotic into necrotic cell death. It should be realized that apoptosis and necrosis are not mutually exclusive, but share identical intracellular signaling pathways. Hence, the term necroapoptosis has been adopted to describe the fact that necrosis and apoptosis are two sides of the same coin (Nieminen 2003; Nikoletopoulou et al. 2013; Stevens et al. 2013). Indeed, Hong et al. (2009) reported that oxidant stress generated by O_2_^•−^ elicits mainly necrotic cell death in an in vivo model of liver injury. The explanation for this difference between in vivo and in vitro models has not been elucidated yet, but may be related to the difference between a culture model and an in vivo model in which the complex microenviroment, composed of additional cell types, hepatic sinusoids, hypoxia, etc. may also play a role (Hong et al. 2009). In contrast, the insulin-sensitizing drug rosiglitazone did not show any protective effect against oxidative stress-induced apoptosis, but rather appeared to aggravate the effect of oxidative stress. Although metformin slightly but significantly increased insulin-induced phosphorylation of IRS-1, insulin itself and glitazones do not protect against oxidative stress-induced cell death. We previously reported that metformin did induce the phosphorylation of its down-stream target AMPK, demonstrating that metformin is performing as expected in our hepatocyte culture system (Woudenberg-Vrenken et al. 2013). These results indicate that the mechanisms of action metformin and rosiglitazone are different and that the protective effect of metformin is independent of insulin signaling. This is supported by the fact that all our experiments were performed in the absence of insulin (unless otherwise indicated).

Previously, we reported that oxidative stress-induced apoptosis is dependent on JNK pro-apoptotic activity, since inhibition of JNK activity, using the inhibitor SP600125, prevents menadione-induced caspase activation and apoptosis in primary hepatocytes (Conde de la Rosa et al. 2006, 2008). Concerning the involvement of JNK in the protective effect of metformin against oxidative stress-induced apoptosis, we demonstrate that metformin reduces JNK-phosphorylation and its pro-apoptotic activity. Therefore, our results indicate that the protective effect of metformin against oxidative stress-induced apoptosis is at least in part via inhibition of JNK activation.

JNK has been described to be a crucial mediator in insulin resistance and a critical element in the pathogenesis of fatty liver disease and type 2 diabetes (Hirosumi et al. 2002; Wellen and Hotamisligil 2005). JNK activity has been reported to be highly elevated in diabetes, due to the presence of inflammation (TNF-*α*), free fatty acids, and oxidative stress, all features associated with diabetes and obesity. JNK can directly phosphorylate IRS-1 inducing defects in insulin signaling and consequently insulin resistance (Aguirre et al. 2000). Thus, it has been reported that JNK-deficient obese mice are protected against insulin resistance and defective insulin receptor signaling (Hirosumi et al. 2002). In addition, dominant-negative JNK isoform in the livers of obese animal results in increased insulin sensitivity and glucose homeostasis. Furthermore, exogenous expression of JNK in adult liver results in insulin resistance in mice (Nakatani et al. 2004). Metformin-induced inhibition of JNK phosphorylation could be related to its well-known properties as an insulin sensitizing agent.

However, in our study, experiments were performed in the absence of insulin. Therefore, it is unlikely that the protective effect of metformin is related to early events in insulin signal transduction. Nevertheless, it cannot be excluded that metformin interferes with down-stream steps of insulin signal transduction, for example, inhibition of JNK activation.

The mechanisms by which JNK exert its pro-apoptotic properties are not completely understood. In many studies, an effect of JNK has been described at the level of the mitochondria triggering the mitochondrial death pathway, including phosphorylation and activation of pro-apoptotic bcl-2 family members (Czaja et al. 2003; Tournier et al. 2000; Okuno et al. 2004; Lei et al. 2002; Chauhan et al. 2003). Our results correlate with a previous study in vivo, in which basal protein expression of JNK was reduced after metformin treatment in Wistar rats (Cleasby et al. 2004).

HO-1 has been shown to be protective in many studies (Amersi et al. 1999; Otterbein et al. 1999; Rensing et al. 1999; Kyokane et al. 2001; Chiu et al. 2002; Sass et al. 2003; Dorman et al. 2004; Ryter et al. 2006). Previously, we demonstrated that overexpression of HO-1, using the adenovirus Ad5HO-1, leads to protection against oxidative stress (menadione)-induced apoptosis in primary hepatocytes (Conde de la Rosa et al. 2008). In this report, we document that metformin further increases menadione-induced HO-1 expression. Metformin alone does not induce HO-1 expression in primary hepatocytes. Experiments using transition metallo-porphyrins as HO-1 inhibitors to confirm the protective effect of HO-1 failed in our hands, due to strong toxic effects of these compounds. A potential mechanism for metformin-induced HO-1 expression may be related to hydrogen sulfide (H_2_S). It has been shown that hydrogen sulfide induces HO-1 expression (D'Araio et al. 2014). Interestingly, metformin has been reported to increase hydrogen sulfide levels (Wilinski et al. 2013) and we have preliminary data that hydrogen sulfide-generating enzymes are present in hepatocytes. All in all, these results suggest that the increase of HO-1 expression by metformin contributes to the protection against oxidative stress-induced apoptosis.

This is the first time that a study shows the involvement of HO-1 induction and JNK inhibition in the protective effect of metformin against oxidative stress-induced apoptosis. In our studies, we demonstrated the protective effect of HO-1 against oxidative stress-induced apoptotic cell death. However, HO-1 also protects against necrotic cell death in an in vivo model of acetaminophen overload (Chiu et al. 2002; Dorman et al. 2004). Since hepatocyte death in liver disorders most likely has both a necrotic and apoptotic component, HO-1 induction, and hence metformin, could be beneficial in liver disorders accompanied by extensive apoptotic and/or necrotic hepatocyte death. Increased HO-1 expression has also been shown to correlate with increased expression of anti-apoptotic bcl-2 family members like bcl-xl (Goodman et al. 2007; Banerjee et al. 2012). Indeed, we demonstrate in this study that bcl-xl expression correlates with HO-1 expression and reaches the highest level in response to the combination metformin and oxidative stress. The concentrations of metformin used in this study are high in comparison to the plasma levels achieved during therapeutic use of metformin, although they are in line with metformin concentrations used in many published in vitro studies. However, our study is important as a ‘proof of concept’ and could lead to clinical application, e.g., using metformin analogs or other agents that induce HO-1 and/or blc-xl expression. Furthermore, metformin in high concentrations could be added to preservation/perfusion solutions of donor organs to protect transplanted organs from oxidative stress-mediated reperfusion injury.

In conclusion, in this study, we demonstrate the protective role of metformin against oxidative stress-induced apoptosis in primary hepatocytes. In addition, we report a completely novel mechanism by which metformin may protect against apoptosis, involving the induction of HO-1 and bcl-xl expression and the reduction of JNK activation. Due to the central role of JNK in pathogenesis of oxidative stress-mediated diseases, including diabetes and most chronic liver diseases, modulation of JNK activity represents a promising and attractive target for the treatment of these disorders.

## Conflict of Interest

The authors declare that they have no conflict of interest with other people or organizations within 3 years of beginning the work submitted that could inappropriately influence the work submitted.

## Abbreviations

H_2_S: hydrogen sulfide
SDS-PAGE: sodiumdodecyl sulphate polyacrylamide gel electrophoresis
ROS: reactive oxygen species
O_2_^•−^: superoxide anions
H_2_O_2_: hydrogen peroxide
HO-1: heme oxygenase-1
PARP: poly(ADP-ribose) polymerase
MAPK: mitogen-activated protein kinase
JNK: c-Jun N-terminal kinase
GAPDH: glyceraldehyde 3-phosphate dehydrogenase
IRS-1: insulin receptor substrate-1
NAFLD: nonalcoholic fatty liver disease
NASH: nonalcoholic steatohepatitis
